# Suppression of Hepatic Epithelial-to-Mesenchymal Transition by Melittin via Blocking of TGFβ/Smad and MAPK-JNK Signaling Pathways

**DOI:** 10.3390/toxins9040138

**Published:** 2017-04-13

**Authors:** Ji-Hyun Park, Byoungduck Park, Kwan-Kyu Park

**Affiliations:** 1 College of Pharmacy, Keimyung University, Dalgubeoldaero, Dalseo-Gu, Daegu 42601, Korea; jihyunp@kmu.ac.kr; 2 Department of Pathology, Daegu Catholic University Medical Center, Duryugongwon-ro, Nam-gu, Daegu 42472, Korea

**Keywords:** melittin, epithelial-to-mesenchymal transition, transforming growth factor, liver diseases

## Abstract

Transforming growth factor (TGF)-β1 plays a crucial role in the epithelial-to-mesenchymal transition (EMT) in hepatocytes and hepatic stellate cells (HSC), which contributes to the pathogenesis of liver fibrosis. Melittin (MEL) is a major component of bee venom and is effective in rheumatoid arthritis, pain relief, cancer cell proliferation, fibrosis and immune modulating activity. In this study, we found that MEL inhibits hepatic EMT in vitro and in vivo, regulating the TGFβ/Smad and TGFβ/nonSmad signaling pathways. MEL significantly inhibited TGF-β1-induced expression of EMT markers (E-cadherin reduction and vimentin induction) in vitro. These results were confirmed in CCl_4_-induced liver in vivo. Treatment with MEL almost completely blocked the phosphorylation of Smad2/3, translocation of Smad4 and phosphorylation of JNK in vitro and in vivo. Taken together, these results suggest that MEL suppresses EMT by inhibiting the TGFβ/Smad and TGFβ/nonSmad-c-Jun N-terminal kinase (JNK)/Mitogen-activated protein kinase (MAPK) signaling pathways. These results indicated that MEL possesses potent anti-fibrotic and anti-EMT properties, which may be responsible for its effects on liver diseases.

## 1. Introduction

Transforming growth factor (TGF)-β expression is increased markedly in the cirrhotic liver and is a potent inducer of stellate cell proliferation and collagen production [1]. In the long pathological period between hepatic fibrosis to cirrhosis, TGF-β1 has been considered as one of the strongest pro-fibrotic cytokines [2,3] and the TGF-β signaling pathway is the cardinal signal transduction pathway, as verified by previous studies [4]. TGF-β is a growth factor that regulates many cellular processes, including proliferation, differentiation, apoptosis and epithelial-to-mesenchymal transition (EMT) [5]. TGF-β1-induced EMT has been implicated in hepatic fibrosis, cirrhosis, and tumor metastasis [6]. TGF-β can induce typical EMT in culture hepatocytes, including primary hepatocytes, AML12 murine hepatocytes and hepatic stellate cells [7]. Recently, evidence that TGF-β1 turned adult mouse hepatocytes into activated fibroblasts through the EMT process, which contributes to liver fibrosis, was reported [8,9].

Liver fibrosis results from increased deposition of type-I collagen within the hepatic extracellular space and constitutes a common signature of all forms of liver injury irrespective of etiology [10]. Excessive extracellular matrix (ECM) deposition, produced by hepatic myofibroblasts without sufficient degradation, results in destruction and distortion of normal hepatic architecture, which is the major pathological progress of the development of liver fibrosis and cirrhosis [11]. Although hepatic stellate cells (HSC) activated by chronic inflammation have been considered as key players in the hepatic fibrogenic process [12,13], other cell types, either hepatic (i.e., portal fibroblast, hepatocyte) or extrahepatic (bone marrow-derived cells, circulating fibrocytes) could also contribute to this process. The contribution of EMT of hepatocytes to the fibrogenic process has been emerging as an important research topic in hepatic fibrogenesis [14].

Bee venom of *Apis mellifera* is a traditional Korean medicine that has been widely used with satisfactory results in the treatment of some immune-related diseases [15]. Melittin (MEL) is a hemolytic peptide that is the major component in the bee venom [16]. MEL has multiple effects including antibacterial, antiviral, anticancer and anti-inflammatory activities in various cell types. These diverse activities of MEL have been comprehensively reviewed [17]. We recently demonstrated that MEL efficiently suppresses specific gene expression in apoptotic liver failure, liver inflammation, xenobiotic-induced cholestasis, atherosclerosis and *Propionibacterium acnes* induced inflammatory responses in vitro and in vivo [16,18,19,20,21]. Also, we reported that MEL protects against TGF-β1-induced hepatocyte cell death by inhibiting apoptosis signaling [22]. However, these studies are insufficient to demonstrate that MEL can prevent EMT in vitro and in vivo. Therefore, we further evaluated the molecular mechanism underlying the anti-EMT activities of MEL by observing its effect in vitro and in vivo. We demonstrated that MEL suppressed EMT by affecting multiple TGF-β1-mediated molecular mediators involved in liver injury.

## 2. Results

### 2.1. Effects of MEL on TGF-β1-Induced EMT In Vitro

**Figure 1:**
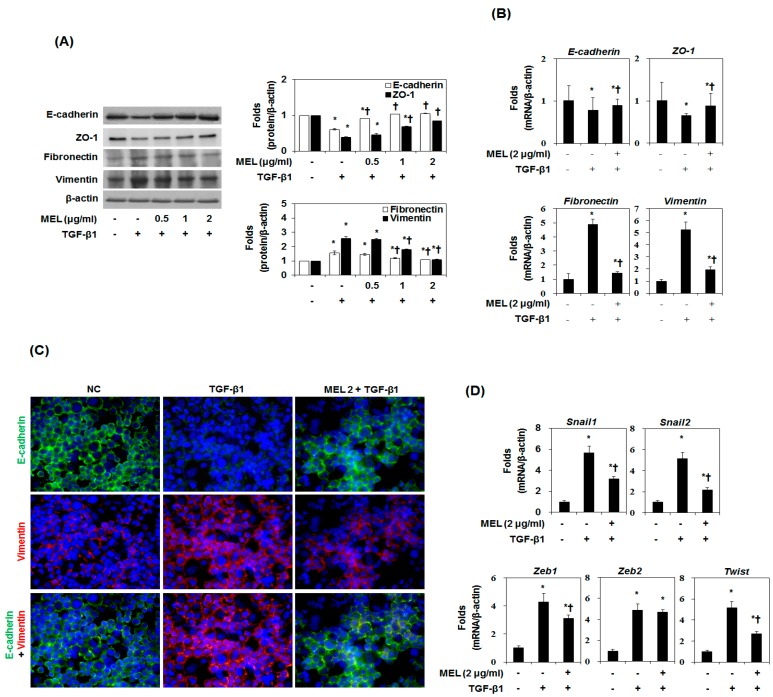
Effects of melittin (MEL) on TGF-β1-induced epithelial-to-mesenchymal transition (EMT) in vitro. Cells were pretreated for 1 h with MEL, followed by incubation with TGF-β1 for 48 h. (**A**) MEL inhibited the TGF-β1-stimulated EMT marker in AML12. The quantitative ratios are shown as relative optical densities of bands that are normalized to the expression of β-actin; (**B**) E-cadherin, ZO-1, fibronectin and vimentin mRNA expression was analyzed by real-time PCR in vitro. The quantitative ratios are shown as relative optical density that are normalized to the expression of β-actin; (**C**) Immunofluorescence double staining for E-cadherin (green) and vimentin (red) in TGF-β1-stimulated AML12 after treatment of MEL. Cells was counterstained with Hoechst 33342 (blue). Magnifications ×200; (**D**) Real-time PCR of EMT related gene markers. The quantitative ratios are shown as relative optical density that are normalized to the expression of β-actin. The data are representative of three similar experiments and quantified as mean values ± S.E. * *p* < 0.05 versus normal control, † *p* < 0.05 versus TGF-β1 treatment.

TGF-β1-induced EMT was performed using the cultured hepatocyte AML12 and HSC according to a method described previously [23]. In response to TGF-β1, both AML12 and HSC acquired a spindle-like mesenchymal morphology, which could be detected after 12 h and became prominent after 48 h (Figure S1a). In the EMT process of hepatocytes, E-cadherin is downregulated and vimentin is upregulated [23]. We determined the expression of EMT markers in TGF-β1-stimulated AML12 and HSC. TGF-β1 treatment stimulated type-I collagen and α-SMA expression time-dependently in AML12 and HSC (Figure S1b). In addition, downregulation of E-cadherin and upregulation of vimentin occurred during TGF-β1 treatment in a time-dependent manner. To investigate the effect of MEL in TGF-β1-EMT progression, AML12 was pretreated with MEL for 1 h and then stimulated with TGF-β1 for 48 h (Figure 1A). MEL treatment resulted in cellular resistance to expression of EMT markers (epithelial marker: E-cadherin and ZO-1, mesenchymal marker: fibronectin and vimentin) in a dose-dependent manner. The suppressive effect on the expression of markers for EMT by MEL 2 μg/mL was confirmed by real-time PCR (Figure 1B). We also carried out immunofluorescence staining to examine the expression of E-cadherin and vimentin in AML12. As shown in Figure 1C, MEL abrogated downregulation of E-cadherin and upregulation of vimentin expression. Consistent with these results, TGF-β1 treatment reduced membrane-associated expression of E-cadherin, with a loss of expression at cell borders and concomitant increases in vimentin expression in a fibril-associated pattern. AML12 concurrently treated with MEL and TGF-β1 maintained high levels of localized expression of E-cadherin; they also showed no increase in the levels of mesenchymal markers. Candidate E-cadherin transcription repressors are members of the Snail, Twist and ZEB families [24]. Therefore, we determined whether treatment with MEL impaired the endogenous expression of *Snail1/2*, *Zeb1/2* and *Twist*. As shown in Figure 1D, MEL significantly attenuated the expression of *Snail1/2*, *Zeb1* and *Twist* mRNA in TGF-β1-stimulated AML12. In addition, MET treatment dose-dependently suppressed TGF-β1-induced α-SMA as well as EMT markers expression in HSC protein and mRNA (Figure S2). These results demonstrate that MEL plays an important role in suppressing TGF-β1-induced EMT in vitro.

### 2.2. MEL Prevents EMT via TGF-β/Smad Signal Transduction Pathway

**Figure 2:**
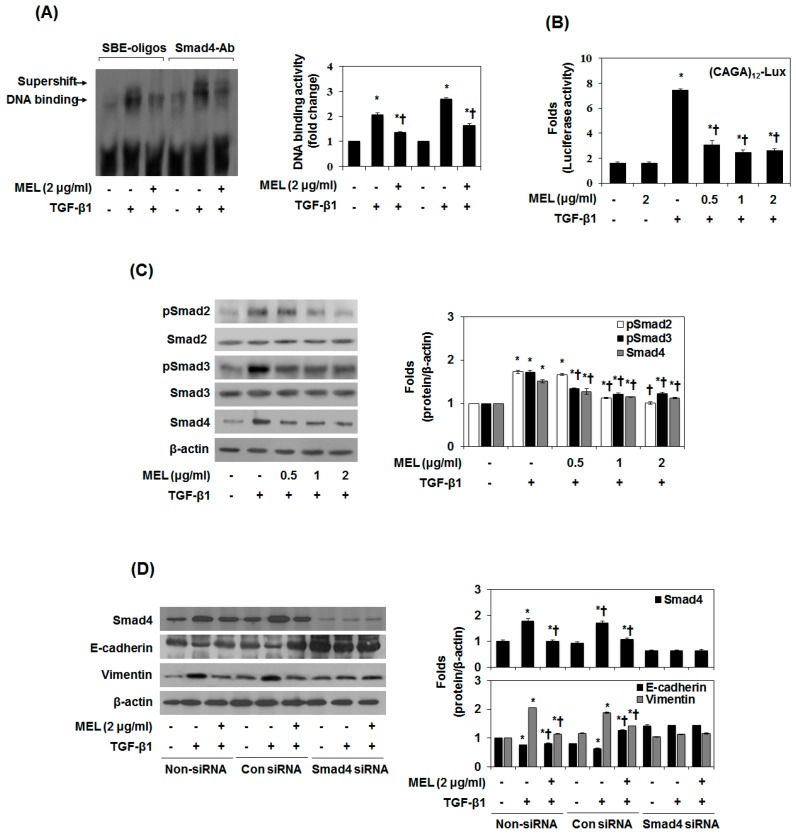
MET antagonizes the TGF-β1-stimulated Smad signal pathway in vitro. Cells were pretreated for 1 h with MEL, followed by incubation with TGF-β1 for 24 h. (**A**) Nuclear extracts were subjected to SBE DNA binding and Smad4 antibody (Ab) assay by EMSA supershift assay; (**B**) MEL inhibits TGF-β1-dependent transcriptional activity of the CAGA_x12_-Luc reporter in a dose-dependent manner; (**C**) Immunoblot of the effect of MEL on the TGF-β1-stimulated pSmad2, pSamd3 and Smad4; (**D**) AML12 was transfected with control (Con) or specific Smad4 siRNA and then treated with TGF-β1 for 24 h or 48 h. The quantitative ratios are shown as relative optical densities of bands that are normalized to the expression of β-actin. The data are representative of three similar experiments and quantified as mean values ± S.E. * *p* < 0.05 versus normal control, † *p* < 0.05 versus TGF-β1 treatment.

In TGF-β1-mediated EMT, the phosphorylated Smad3/4 complex activates the transcription of TGF-β1-mediated genes through interactions with other DNA-binding transcription factors [25]. To determine whether MEL antagonizes the effects of TGF-β1 by interfering with Smad3/4 binding to DNA, we performed an EMSA and supershift assay using a SBE-specific sequence and Smad4 Ab complexes. SBE-DNA and Smad4-Ab complexes were strongly increased by TGF-β1, and this increase was inhibited by MEL (Figure 2A). The inhibitory effect of MEL on TGF-β1-dependent gene transcription of the (CAGA)_12_-Lux reporter was observed in nature (Figure 2B). Moreover, MEL abrogated the TGF-β1-mediated phosphorylation of Smad2/3 in a dose-dependent manner (Figure 2C). Upon phosphorylation, Smad2/3 forms a complex with the co-mediator Smad4 and subsequently translocate into the nucleus. Within the nucleus, the Smad2/3/4 complex regulates the transcription of target genes responsible for EMT [26]. We examined the role of TGF-β1/Smad in EMT regulation by pretreating AML12 with Smad4 siRNA. After transfection with Smad4 siRNA for 24 h, the expression of Smad4 was declined in TGF-β1-stimulated AML12 (Figure 2D and Figure S3a). However, non-siRNA and transfection with control siRNA did not affect Smad4 accumulation. Furthermore, MEL and Smad4 siRNA markedly increased E-cadherin expression and reduced vimentin expression in TGF-β1-stimulated AML12. Taken together, these results suggest that MEL ameliorates TGF-β1-mediated EMT by blocking the TGF-β1/canonical Smad pathway.

### 2.3. MEL Prevents EMT via a TGF-β/Non-Smad-Dependent Pathway

The induction of EMT by TGF-β1 is associated with the activation of Mitogen-activated protein kinases (MAPKs), PI3K/Akt and RhoA [27]. TGF-β1 exerts pro-fibrotic effects through activation of c-Jun N-terminal kinase (JNK) signaling pathways [23]. JNK activation positively regulates the expression of Snail and Slug [28].

**Figure 3:**
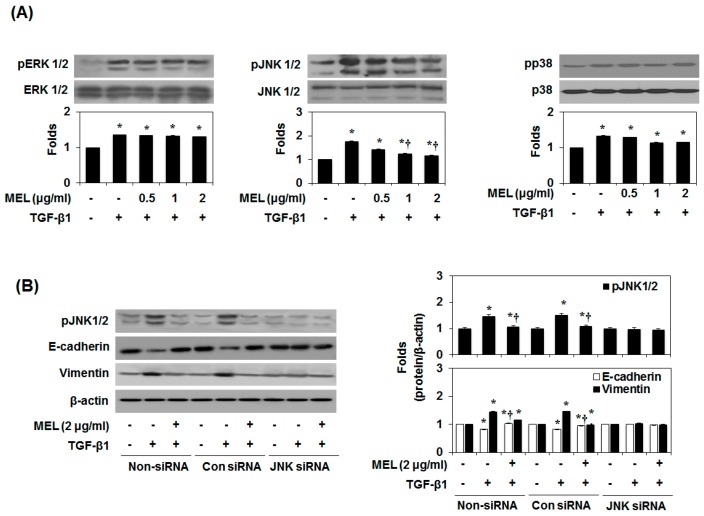

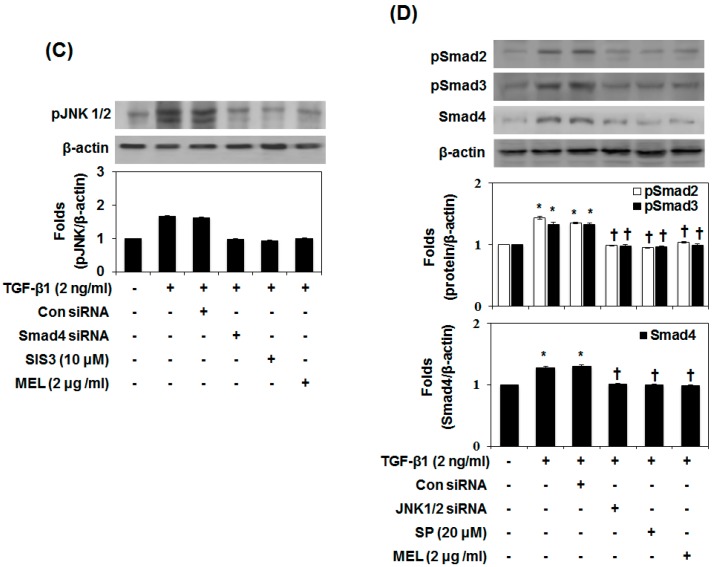
MEL antagonizes the TGF-β1-stimulated non-Smad/MAPK signal pathway. (**A**) Immunoblot of mitogen-activated protein kinase (MAPK). MEL strongly inhibited the TGF-β1-stimulated pJNK1/2; (**B**) AML12 was transfected with control (Con) or specific c-Jun N-terminal kinase (JNK)1/2 siRNA and then treated with TGF-β1 for 24 h or 48 h; (**C**) Smad4 siRNA, Smad3 inhibitor (SIS) and MEL inhibited TGF-β1-stimulated pJNK1/2 expression; (**D**) JNK siRNA, JNK inhibitor (SP, SP600125) and MEL inhibited TGF-β1-stimulated Smad4 expression. The quantitative ratios are shown as relative optical densities of bands that are normalized to the expression of β-actin. The data are representative of three similar experiments and quantified as mean values ± S.E. * *p* < 0.05 versus normal control, † *p* < 0.05 versus TGF-β1 treatment.

Therefore, we aimed to identify the MAPK signaling pathway that affects the inhibition by MEL of TGF-β1-induced EMT. As shown in Figure 3A, MEL strongly abrogated the TGF-β1-mediated phosphorylation of JNK1/2 in a dose-dependent manner. Subsequently, we confirmed the JNK1/2 MAPK-dependent expression of E-cadherin and vimentin by pre-treating AML12 with JNK1/2 siRNA. After transfection with JNK1/2 siRNA for 24 h, JNK1/2 expression decreased in TGF-β1-stimulated AML12 (Figure 3B and Figure S3b). However, non-siRNA and transfection with control siRNA did not affect JNK1/2 expression. Furthermore, JNK1/2 siRNA and MEL most strongly increased E-cadherin expression and reduced the expression of vimentin in TGF-β1-stimulated AML12. To further investigate the role of Smad4 and JNK-MAPK in TGF-β1-induced phosphorylation of JNK1/2, Smad2 and Smad3, and expression of Smad4, we examined the effect of Smad4 siRNA, JNK1/2 siRNA and inhibitors by immunoblotting. As shown in Figure 3C, TGF-β1-induced phosphorylation of JNK1/2 was inhibited by Smad4 siRNA, inhibitors and MEL. Moreover, TGF-β1-induced phosphorylation of Smad2 and Smad3 and expression of Smad4 were inhibited by JNK1/2 siRNA, inhibitors and MEL (Figure 3D). These results demonstrate that MEL plays an important role in suppressing TGF-stimulated EMT, and that this activity is mediated through the JNK-MAPK signaling pathway in vitro.

### 2.4. MEL Ameliorates Liver EMT Induced by CCl_4_

We previously reported a liver fibrosis model established by chronic CCl_4_ injection [29]. The morphological and histological changes in liver fibrosis induced by CCl_4_ were visualized in sections stained with H&E and Masson’s trichrome. According to the histological analysis, the control liver showed a normal lobular architecture with central veins and radiating hepatic cords. After eight weeks of CCl_4_ induction, the increase and expansion of fibrous septae, ballooning changes of hepatocytes and multifocal hepatocellular necrosis were remarkable. Deposition of collagen fibers was evident by trichrome staining (Figure S4a). Immunofluorescence staining was carried out to examine the localization and expression of E-cadherin and vimentin. CCl_4_ induction reduced the membrane-associated expression of E-cadherin with loss of expression at cell borders and concomitant increases in vimentin expression in a fibril-associated pattern. In agreement with the immunoblotting results, CCl_4_ induction markedly induced TGF-β1 expression for eight weeks (Figure S4b). During CCl_4_ induction, E-cadherin was downregulated seven-fold and vimentin was upregulated two-fold compared to NC group.

**Figure 4:**
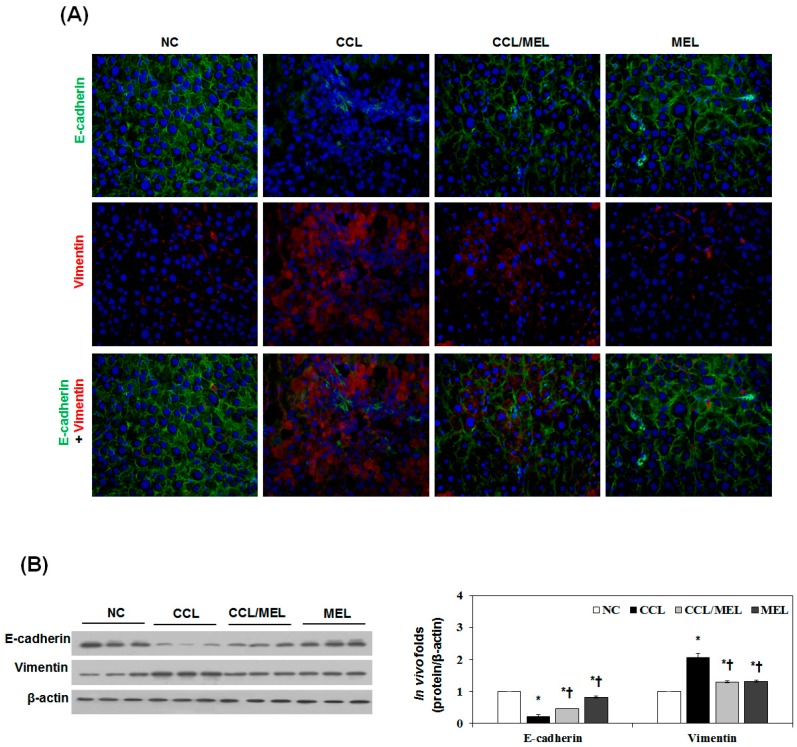
MEL suppressed CCl_4_-induced EMT in vivo. (**A**) Immunofluorescence double staining for E-cadherin (green) and vimentin (red) in CCl_4_ induced changes in EMT markers. Cells was counterstained with Hoechst 33342 (blue). Magnifications ×200; (**B**) Immunoblot results show the effects of MEL on the inhibition of CCl_4_ induced changes in EMT markers including E-cadherin and vimentin. The quantitative ratios are shown as relative optical densities of bands that are normalized to the expression of β-actin. The data are representative of three similar experiments and quantified as mean values ± S.E. * *p* < 0.05 versus NC, † *p* < 0.05 versus CCL.

To determine whether MEL inhibits CCl_4_-induced EMT, tissue sections of cirrhotic liver were examined by immunhistopathology, immunohistochemistry, immunoblotting and real-time PCR. As shown in Figure 4A, the NC group demonstrated expression of E-cadherin localized at the cell border and low levels of vimentin expression. However, the CCL group exhibited loss of E-cadherin at cell-cell junctions, accompanied by increased expression of vimentin. The CCL/MEL group maintained high levels of localized E-cadherin expression and showed no increase in vimentin. The effect of MEL on E-cadherin and vimentin expression was also confirmed by immunoblotting (Figure 4B). Consistent with the immunofluorescence staining, immunoblotting analysis showed decrease of E-cadherin and increase of vimentin expression in the CCL group compared with the NC group. In contrast, the CCL/MEL group exhibited significant inhibition of the decreased E-cadherin and increased vimentin expression.

### 2.5. MEL Ameliorates EMT through TGFβ/Smad and JNK-MAPK In Vivo

**Figure 5:**
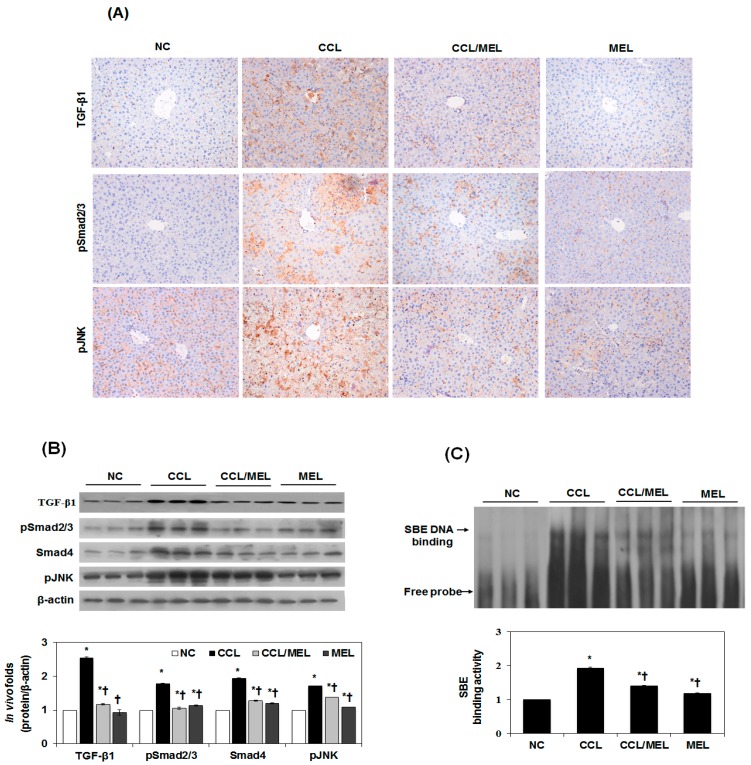
Effect of MEL on Smad and non-Smad/MAPK signal pathway in vivo. (**A**) Typical examples of immunohistochemical staining of TGF-β1, pSmad2/3 and pJNK; (**B**) Immunoblot show that inhibition of TGF-β1, pSmad2/3, Smad4 and pJNK expression by MEL. The quantitative ratios are shown as relative optical densities of bands that are normalized to the expression of β-actin; (**C**) Nuclear extracts were subjected to SBE DNA binding assay by EMSA. The data are representative of three similar experiments and quantified as mean values ± S.E. * *p* < 0.05 versus NC, † *p* < 0.05 versus CCL.

We have determined the expression of TGFβ/Smad and JNK-MAPK in vivo in accordance with the in vitro results. As shown in Figure 5A, TGF-β1, psmad2/3 and pJNK1/2 were significantly increased in the CCL group compared to the NC group. However, this increase was attenuated in the CCL/MEL group. The suppressive effect on psmad2/3, Smad4, and pJNK1/2 expression by MEL was confirmed by immunoblotting (Figure 5B). In agreement with the immunohistochemistry results, the CCL/MEL group exhibited significantly inhibited psmad2/3 and pJNK1/2 and Smad4 expression. In addition, Smad3/4-DNA-binding activity was significantly increased in the CCL group compared to the NC group, and this increase was inhibited in the CCL/MEL group (Figure 5C). These results indicated that MEL abrogated EMT through the TGFβ/Smad and JNK-MAPK signaling pathways.

## 3. Discussion

Liver fibrosis, which results from overproduction of ECM proteins such as collagens and TGF-β1, precedes the development of cirrhosis, which is an end-stage consequence of severe liver damage secondary to various types of chronic liver disease [30]. EMT plays an important role in liver fibrosis [16,31]. Recently, we reported that MEL prevents thioacetamide-induced liver inflammation by interrupting the NF-κB signaling pathway [19]. Our previous reports showed that MEL inhibited hepatocyte death via inhibition of apoptosis signaling in vitro and in vivo [18,22]. However, these studies are insufficient to demonstrate that MEL can prevent the development of EMT in vitro and in vivo.

Studies of EMT and apoptosis by TGF-β1 are of great importance for understanding the mechanisms underlying the progress of liver cirrhosis [32]. We reported previously that MEL prevents mitochondria-mediated apoptosis in vitro [22]. Also, we recently demonstrated that MEL suppresses galactosamine/LPS-stimulated hepatic failure through the mitochondria-dependent apoptosis and inflammatory signaling pathways [18]. In the present study, we found that MEL potently attenuated markers for EMT in vitro model induced by TGF-β1 and in vivo model induced by CCl_4_.

TGF-β1 exerts its effects via Smad-dependent activation and Smad-independent induction of MAPK pathways [33]. The TGFβ receptor (TβR)I and TβRII are transmembrane Ser/Thr kinase receptors that phosphorylate the receptor-associated Smad proteins Smad2/3. Once phosphorylated, the heterotrimeric complex of Smad3/4 enters the nucleus and activates the expression of target genes [34]. In the present in vitro and in vivo study, MEL potently attenuated Smad3/4 complex nuclear translocation and TGF-β1-dependent transcription of the (CAGA)_12_-Lux reporter in vitro. Also, MEL significantly inhibited phosphorylation of Smad2/3 in vitro and in vivo.

JNK is phosphorylated during TGF-β1-induced hepatic fibrosis and EMT [35], and JNK activity is required for disassembly of adherent junctions and induction of cell motility, and blockade of JNK inhibited key morphological features of EMT [36,37]; hence the inhibition of JNK phosphorylation is a potential target for suppressing liver fibrosis. In agreement with these results, MEL significantly abrogated the phosphorylation of JNK1/2 in vitro model induced by TGF-β1 and in vivo model induced by CCl_4_.

In conclusion, our data demonstrated that MEL inhibits TGFβ/Smad and JNK-MAPK signaling, and suppresses EMT progression in vitro and in vivo. These results suggest that MEL should be considered as novel therapeutic agent for liver fibrosis.

## 4. Materials and Methods

### 4.1. Cell Cultures and Reagents

A non-tumorigenic mouse hepatocyte cell line, AML12 (America Tissue Culture Collection, CRT-2254; ATCC, Manassas, VA, USA), was cultured in a 1:1 mixture of Dulbecco’s Modified Eagle’s Medium/Ham’s F-12 medium (Gibco, Grand Island, NY, USA) containing 5 µg/mL ITS premix (Sigma-Aldrich, St. Louis, MO, USA), 40 ng/mL dexamethasone (Sigma-Aldrich), and 10% fetal bovine serum (FBS, Gibco). According to our previous studies, primary culture rat HSC was cultivated at 37 °C in an atmosphere of 5% CO_2_ in minimum essential medium, supplemented with 10% fetal bovine serum, 100 U/mL penicillin and 100 mg/mL streptomycin (Gibco) [19]. TGF-β1 was obtained from R&D System Inc. (R&D System Inc, Minneapolis, MN, USA). Signal inhibitors were obtained from Calbiochem (Calbiochem, Cambridge, MA, USA). All chemicals were obtained from Sigma-Aldrich including melittin (MEL), unless otherwise indicated.

### 4.2. Animal and Induction of Liver Injury

Male C57BL/6 mice (5–6 weeks old), weighing 20–25 g, were randomly divided into four groups of five mice per group: a normal control group (NC), a CCl_4_-liver damage group (CCL), a CCl_4_-liver damage treated with MEL group (CCL/MEL), MEL treated group (MEL). All experimental protocols were approved by the Institutional Animal Care and Use Committee of the Catholic University of Daegu (Daegu, South Korea, EXP-IRB number: DCIAFCR-151007-8-Y, The date of approval: 7 October 2015) in accordance with criteria outlined in the Institutional Guidelines for Animal Research. All mice were cultured under a 12:12 h light/dark cycle at 25 ± 1 °C and with a controlled humidity of 60% ± 5%.

The chronic liver damage received intraperitoneal injection of CCl_4_ (2 mg/kg) dissolved in corn oil (1:3 ration) three times per week for 8 weeks. The CCL+MEL and MEL groups received intraperitoneal injection of MEL (0.1 mg/kg) dissolved in saline twice a week. Mice were sacrificed after 8 weeks from the first CCl_4_ administration. The mice were euthanized under ether anesthesia.

### 4.3. Morphological Examination

Morphological changes in the cells were observed under a Leica DMIL LED phase contrast microscope with an attached EC3 camera (Leica, Germany). The photographs were taken at 200× magnification.

### 4.4. Protein Isolation and Immunoblot Analysis

Mitochondrial and cytosolic protein fractions were obtained as previously described [25]. The protein concentration was determined with a Bio-Rad Bradford kit (Bio-Rad Laboratories, Hercules, CA, USA). The samples were boiled for 5 min and equal volumes were loaded on a sodium dodecyl sulfate-polyacrylamide gel electrophoresis (SDS-PAGE). After separation, the proteins were transferred to a nitrocellulose membrane during 1 h at 4 °C and blocked overnight with PBS-T (0.1% (*v*/*v*) Tween-20, 5% (*w*/*v*) powdered milk in 137 nm NaCl, 2.7 mM KCl, 1.5 mM KH_2_PO_4_, pH 7.4) at 4 °C. Immune complexes were detected with a horseradish peroxidase (HRP)-conjugated secondary antibody and were visualized by an enhanced chemiluminescence (ECL) detection system (Bio-Rad Laboratories). Specific antibodies for TGF-β1, E-cadherin, vimentin, α-SMA, ZO-1, fibronectin, smad2, psmad2, smad3, psmad3, psmad2/3, smad4, extracellular signal-regulated kinase (ERK)1/2, pERK1/2, c-Jun N-terminal kinase (JNK)1/2, pJNK1/2, p38, pp38 and β-actin (Cell signaling Technology, Danvers, MA, USA). The luminescent signals were analyzed using an ImageQuant LAS 4000 Scanner (GE Healthcare, Piscataway, NJ, USA).

### 4.5. Reverse-Transcription and Real-Time PCR

Total RNA was extracted from cells with TRIzol reagent (Invitrogen Co., Grand Island, NT, USA) according to the manufacturer’s instructions. First strand cDNA was synthesized with oligo-d(T) primer and M-MLV reverse transcriptase (Promega, Madison, WI, USA). Real time PCR was performed in a CFX 96 Touch™ detection system (Bio-Rad Laboratories) using SYBR Green PCR Master Mix (Bio-Rad Laboratories). Each measurement was repeated at least in triplicate and the relative quantity of target mRNA was determined using the comparative threshold (C_t_) method by normalizing target mRNA C_t_ values to those for β-actin (ΔC_t_). The optimized primers used for real-time PCR are listed in Table S1.

### 4.6. DNA Transfection and Luciferase Assay

Reporter gene activity was evaluated by cell-based analysis methods for assaying Smad3/4 activity. To measure TGF-β1 signaling the TGF-β1-sensitive reporter construct, 100 ng of (CAGA)_12_-Luc reporter, which encodes 12 copies of the CAGA canonical Smad DNA binding sequence [6]. The cells were cotransfected with plasmid constructs and 0.2 μg of the Renilla reporter plasmid for 6 h using Lipofectamine reagent (Invitrogen, Carlsbad, CA, USA) according to the manufacturer’s protocol. After transfection, cells were pretreated with MEL for 1 h and then stimulated with TGF-β1 for 24 h. Luciferase and Renilla activities were determined by following the manufacturer’s protocol (Dual-Luciferase Reporter Assay System, Promega, Madison, WI, USA).

### 4.7. Transient Transfection with Small Interfering RNA

The cells were transfected with control siRNA (Santa Cruz, CA, USA), JNK1/2-MAPK siRNA and Smad4 siRNA (Cell Signaling Technology, Danvers, MA, USA) using Lipofectamine RNAiMAX transfection reagent (Invitrogen, Carlsbad, CA, USA) according to manufacturer’s instruction. 

### 4.8. DNA Binding Activity of SBE and Smad4 Antibody

According to our previous studies, DIG Gel Shift Kit (Roche, Mannheim, Germany) was performed to detect Smad binding element (SBE) DNA-binding and Smad4 antibody-binding activity, with the instructions of manufacturer [25,38]. The binding activity of Smad 3/4 in vitro and in vivo nuclear extract was confirmed by EMSA or supershift assay with a DIG-labeled oligonucleotide probe (SBE: 5′-TCG AGA GCC AGA CAA TTA GCC-3′—only sense strands are shown) and Smad4 antibody (Cell signaling). EMSA was performed by incubating 10 μg of nuclear extract in a 9 μL binding reaction mixture containing 10 mM Tris–HCl (pH 7.5), 50 mM NaCl, 0.5 mM EDTA, 0.5 mM DTT, 3 mM MgCl_2_, 0.05 mg/mL poly (dI-dC) and 10% (*v*/*v*) glycerol at 37 °C for 10 min. The binding reaction mixture for the super shift assay containing 1 μL of the non-diluted antibody of Smad4 was added to 1 μL of DIG-labeled double-stranded oligonucleotide and was incubated at 37 °C for 20 min, followed by the addition of 1 μL of the gel loading 10× buffer (250 mM Tris–HCl, pH 7.5, 40% glycerol) at room temperature. The DNA-protein complexes were separated by electrophoresis in 6% non-denaturing polyacrylamide gels using 0.25× Tris-borate-EDTA as a running buffer. After electrophoresis, the gels were transferred to nylon membranes and detected chemiluminescent. The luminescent signals were analyzed using an ImageQuant LAS 4000 Scanner of GE Healthcare.

### 4.9. Histopathological Investigation and Immunofluorescence Staining

Liver specimens were fixed overnight in 10% formalin solution, then dehydrated, embedded in paraffin, and cut into 10 μm sections. A cross-section taken from the blocks was stained with hematoxylin and eosin stains (H&E, Sigma-Aldrich, St. Louis, MO, USA) and Masson’s trichrome. For immunohistochemistry, sections were incubated with anti-TGF-β1 (R&D Systems, Minneapolis, MN, USA), psmad2/3 and pJNK (Cell signaling) for 1 h at 37 °C, processed by an indirect immuneperoxidase technique using a commercial kit (DAKO, CA, USA). Light microscopy was used to acquire the immunohistochemical images. The primary antibodies for the cells and tissue section immunofluorescent staining are as follows: E-cadherin and vimentin were purchased form Cell signaling, and secondary antibodies conjugated with Alexa Flour 488 and Alexa Flour 594 were purchased from Invitrogen. Cells were counterstained with Hoechst 33342 (ImmunoChemistry, Bloomington, MN, USA). Slides were mounted using ProLong^®^ Gold antifade reagent (Molecular Probes^®^ by Life Technologies™, Carlsbad, CA, USA). Fluorescence micrographs were acquired with a fluorescence microscope (Nikon ECLIPSE Ti-U, Nikon Co., Tokyo, Japan).

### 4.10. Statistical Analysis

Mean values were used for statistical analyses. Data are expressed as mean ± S.E. For two groups, unpaired two-tailed *t* tests were used; for more than two group comparisons, one-way ANOVAs were used followed by the post hoc Tukey's multiple comparison test; for more than two comparisons of two or more groups, two-way ANOVAs followed by Bonferroni’s post hoc tests were used (Prism GraphPad, Version 5, San Diego, CA, USA). *p* values for the ANOVAs are reported in the figure legends, and *p* values from the post hoc tests are included in the text when making paired comparisons.

## Supplementary Materials

The following are available online at www.mdpi.com/2072-6651/9/4/138/s1, Figure S1: TGF-β1-stimulated EMT in AML12 and HSC. (a) Time effect of TGF-β1 on fibrosis and EMT were examined by morphologic changes in AML12 and HSC, 200× magnification. (b) Expression of fibrosis and EMT markers in TGF-β1–stimulated AML12 and HSC. The quantitative ratios are shown as relative optical densities of bands that are normalized to the expression of β-actin. The data are representative of three similar experiment and quantified as mean values ± S.E. * *p* < 0.05 versus normal control, Figure S2: TGF-β1-stimulated EMT in HSC. MEL inhibited the TGF-β1-stimulated α-SMA and EMT marker in HSC protein expression (a) and RNA expression (b). The quantitative ratios are shown as relative optical densities of bands that are normalized to the expression of β-actin. The data are representative of three similar experiment and quantified as mean values ± S.E. * *p* < 0.05 versus normal control, † *p* < 0.05 versus TGF-β1 treatment, Figure S3: AML12 was transfected with control or specific Smad4 and JNK1/2 siRNA for 24 h. The expression of Smad4 and JNK1/2 were suppressed by Smad4 (a) and JNK1/2 (b) siRNA transfection. The quantitative ratios are shown as relative optical densities of bands that are normalized to the expression of β-actin. The data are representative of three similar experiment and quantified as mean values ± S.E. * *p* < 0.05 versus TGF-β1 treatment, Figure S4: The histological changes in liver fibrosis and EMT induced by CCl_4_. (a) H&E staining (200×), Masson’s trichrome staining (200×), immunohistochemistry for TGF-β1 (400×) and immunofluorescence double staining (400×) for E-cadherin (green) and vimentin (red), × magnification. (b) Expression of fibrosis and EMT markers in CCl_4_ induction for 8 weeks in vivo. The quantitative ratios are shown as relative optical densities of bands that are normalized to the expression of β-actin. The data are representative of three similar experiment and quantified as mean values ± S.E. * *p* < 0.05 versus NC, Table S1: Primers used for real-time qPCR.

## Abbreviations

ECM	Excessive extracellular matrix
HSC	Hepatic stellate cells
EMT	Epithelial-to-mesenchymal transition
TGF	Transforming growth factor
ERK	Extracellular signal-regulated kinase
JNK	c-Jun N-terminal kinase
MAPK	Mitogen-activated protein kinase
LPS	Lipopolysaccharide
TβR	TGFβ receptor
