# Supplementary material for: Suppression of Hepatic Epithelial-to-Mesenchymal Transition by Melittin via Blocking of TGFβ/Smad and MAPK-JNK Signaling Pathways
Source: Toxins (Basel). 2017 Apr 13;9(4):138. doi: 10.3390/toxins9040138 (PMC5408212; doi:10.3390/toxins9040138)
Supplement: Supplementary file 1 [file toxins-09-00138-s001.pdf]

Supplementary Materials: Suppression of hepatic epithelial-to-mesenchymal transition by melittin via blocking of TGFβ/Smad and MAPK-JNK signaling pathways

Ji-Hyun Park, Byoungduck Park and Kwan-Kyu Park

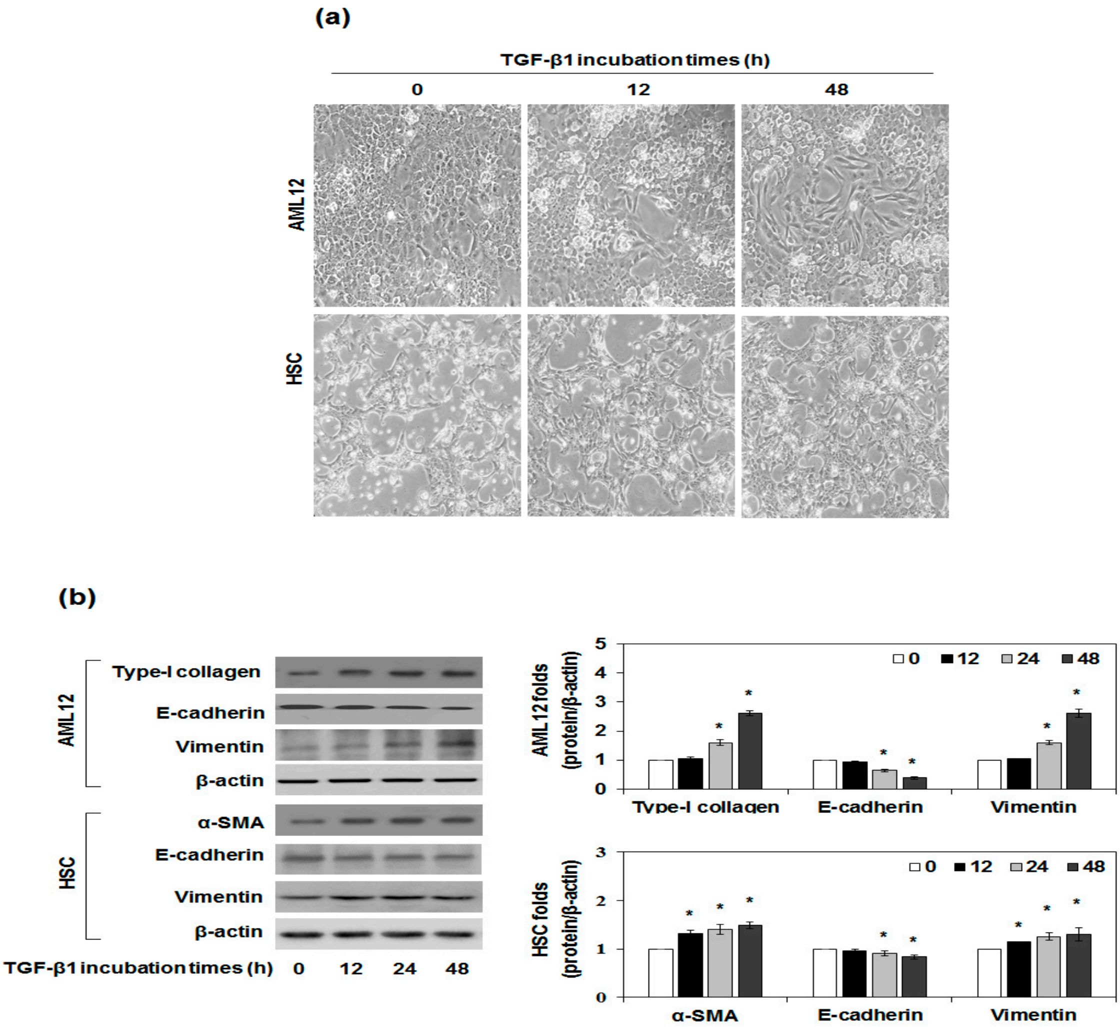

Figure S1. TGF-β1-stimulated EMT in AML12 and HSC. (**a**) Time effect of TGF-β1 on fibrosis and EMT were examined by morphologic changes in AML12 and HSC, 200× magnification. (**b**) Expression of fibrosis and EMT markers in TGF-β1–stimulated AML12 and HSC. The quantitative ratios are shown as relative optical densities of bands that are normalized to the expression of β-actin. The data are representative of three similar experiment and quantified as mean values ± S.E. *$p < 0.05$ versus normal control.

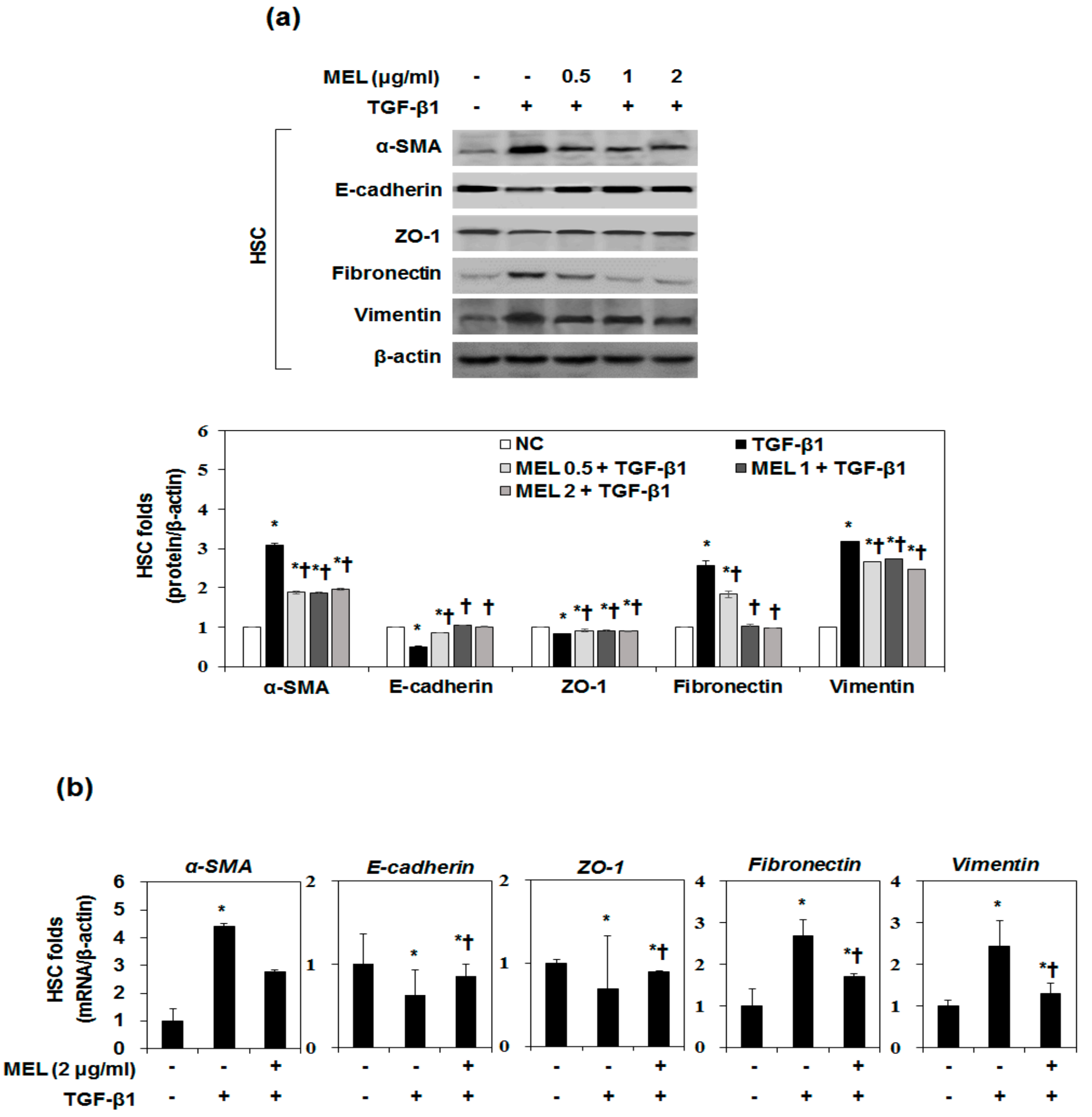

Figure S2. TGF-β1-stimulated EMT in HSC. MEL inhibited the TGF-β1-stimulated α-SMA and EMT marker in HSC protein expression (**a**) and RNA expression (**b**). The quantitative ratios are shown as relative optical densities of bands that are normalized to the expression of β-actin. The data are representative of three similar experiment and quantified as mean values ± S.E. *$p < 0.05$ versus normal control, † $p < 0.05$ versus TGF-β1 treatment.

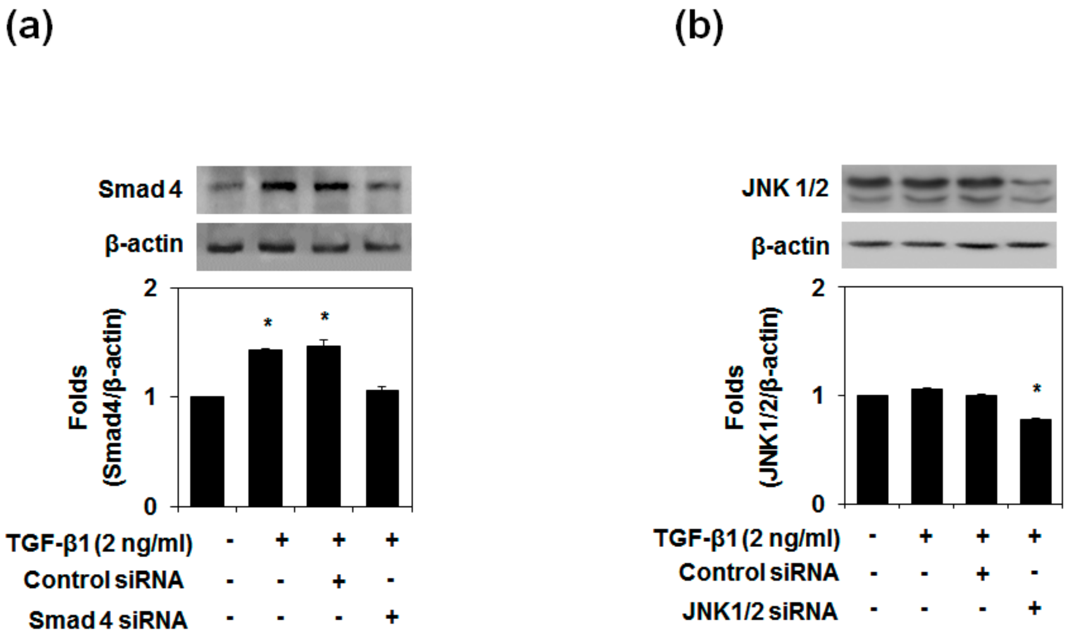

Figure S3. AML12 was transfected with control or specific Smad4 and JNK1/2 siRNA for 24 h. The expression of Smad4 and JNK1/2 were suppressed by Smad4 (**a**) and JNK1/2 (**b**) siRNA transfection. The quantitative ratios are shown as relative optical densities of bands that are normalized to the expression of β-actin. The data are representative of three similar experiment and quantified as mean values ± S.E. *$p < 0.05$ versus TGF-β1 treatment.

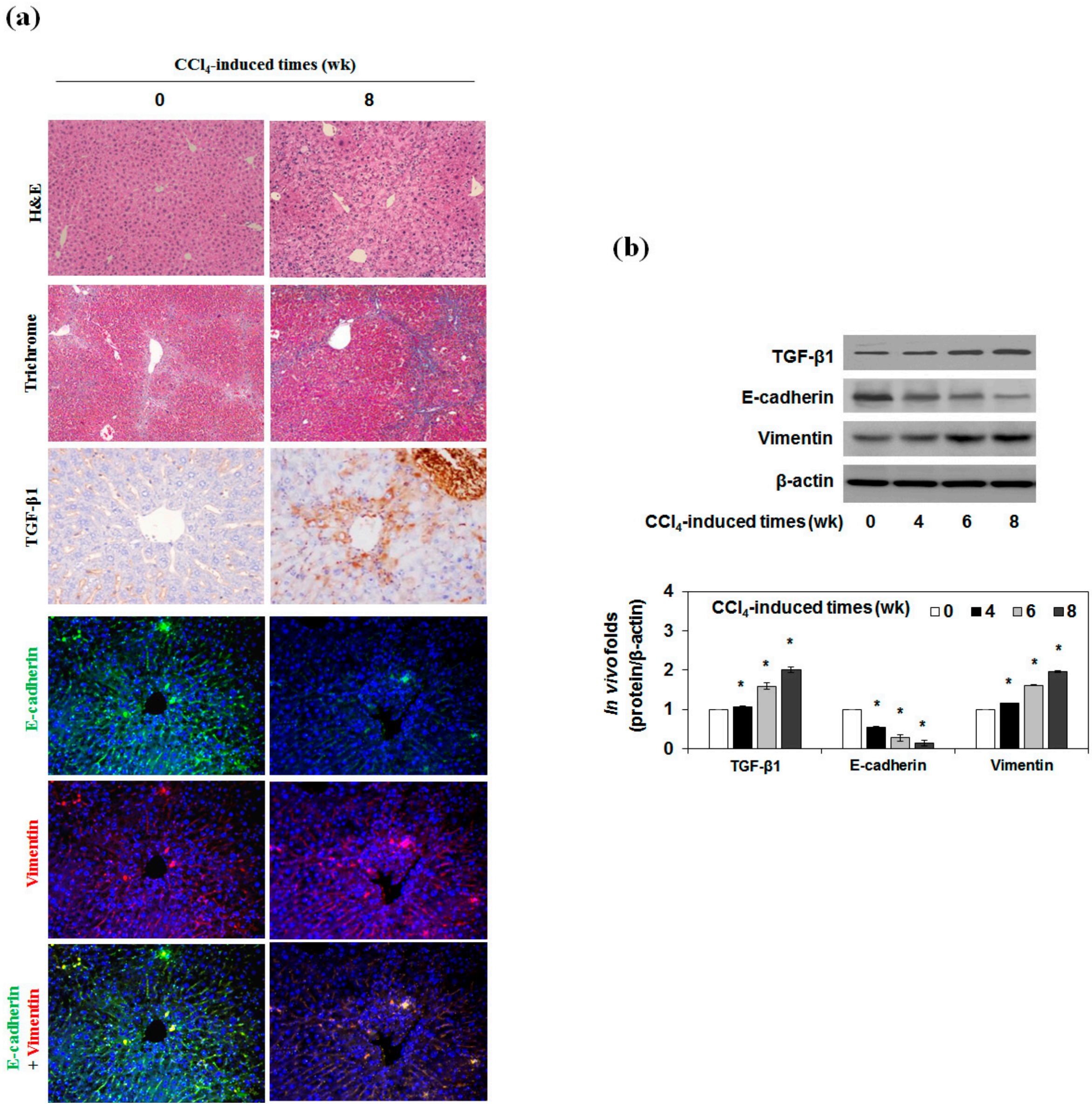

Figure S4. The histological changes in liver fibrosis and EMT induced by CCl_4. (**a**) H&E staining (200×), Masson's trichrome staining (200×), immunohistochemistry for TGF-β1 (400×) and immunofluorescence double staining (400×) for E-cadherin (green) and vimentin (red), × magnification. (**b**) Expression of fibrosis and EMT markers in CCl_4 induction for 8 weeks *in vivo*. The quantitative ratios are shown as relative optical densities of bands that are normalized to the expression of β-actin. The data are representative of three similar experiment and quantified as mean values ± S.E. *$p < 0.05$ versus NC.

Table S1. Primers used for real-time qPCR.

Gene Description	Primer Sequences (F, forward; R, reverse)	References
α-SMA	F: 5′-GACAGAAACGAGACTGGGTCA-3′<br>R: 5′-CCGGTGATGCTGTAGAAAACC-3′	[1]
E-cadherin	F: 5′-GACAGAAACGAGACTGGGTCA-3′<br>R: 5′-CCGGTGATGCTGTAGAAAACC-3′	[2]
ZO-1	F: 5'-ACT CCCACT TCC CCAAAAAC-3'<br>R: 5'-CCACAG CTG AAGGAC TCA CA-3'	[3]
Fibronectin	F: 5′-TCTGGGAAATGGAAAAGGGGAATGG-3′<br>R: 5′-CACTGAAGCAGGTTTCCTCGGTTGT-3′	[1]

Vimentin	F: 5′-GATCGATGTGGACGTTTCCAA-3′<br>R: 5′-GTTGGCAGCCTCAGAGAGGT-3′,	[2]
Zeb1	F: 5′-TGG CAA GAC AAC GTG AAA GA-3′<br>R: 5′-AAC TGG GAA AAT GCT TCT CTG G-3′	[4]
Zeb2	F: 5′-TGA CCG GTC CAG AAG AAA TG-3′<br>R: 5′-GGC CAT CTC TTT CCT CCA GT-3′	[4]
Twist	F: 5′-CCC CAC TTT TTG ACG AAG AAT G-3′<br>R: 5′-AAA ATG GAG CCA GTC ACA TGT GG-3′	[5]
Snail1	F: 5′-CTT GTG TCT CGA CCT GT-3′<br>R: 5′-CTT CAC ATC CGA GTG GGT TT-3′	[6]
Snail2	F: 5′-GCA CTG TGA TGC CCA GTC TA-3′<br>R: 5′-CAG TGA GGG CAA GAG AAA GG-3′	[6]
β-actin	F: 5′- GACCTCTATGCCAACACAGTGC -3′<br>R: 5′-GTACTCCTGCTTGCTGATCCAC-3′	[2]

References

1. Liu, G.; Friggeri, A.; Yang, Y.; Milosevic, J.; Ding, Q.; Thannickal, V.J.; Kaminski, N.; Abraham, E. Mir-21 mediates fibrogenic activation of pulmonary fibroblasts and lung fibrosis. *J. Exp. Med.* **2010**, *207*, 1589–1597.
2. Liu, J.; Zhang, Z.; Tu, X.; Liu, J.; Zhang, H.; Zhang, J.; Zang, Y.; Zhu, J.; Chen, J.; Dong, L., et al. Knockdown of n-acetylglucosaminyl transferase v ameliorates hepatotoxin-induced liver fibrosis in mice. *Toxicol. Sci.: An official journal of the Society of Toxicology* **2013**, *135*, 144–155.
3. Hwang, I.; An, B.S.; Yang, H.; Kang, H.S.; Jung, E.M.; Jeung, E.B. Tissue-specific expression of occludin, zona occludens-1, and junction adhesion molecule a in the duodenum, ileum, colon, kidney, liver, lung, brain, and skeletal muscle of c57bl mice. *J. physiol. and pharmacol.: an official journal of the Polish Physiological Society* **2013**, *64*, 11–18.
4. Oba, S.; Kumano, S.; Suzuki, E.; Nishimatsu, H.; Takahashi, M.; Takamori, H.; Kasuya, M.; Ogawa, Y.; Sato, K.; Kimura, K., et al. Mir-200b precursor can ameliorate renal tubulointerstitial fibrosis. *PloS one* **2010**, *5*, e13614.
5. Copple, B.L. Hypoxia stimulates hepatocyte epithelial to mesenchymal transition by hypoxia-inducible factor and transforming growth factor-beta-dependent mechanisms. *Liver Int.: Official journal of the International Association for the Study of the Liver* **2010**, *30*, 669–682.
6. Chen, Y.L.; Lv, J.; Ye, X.L.; Sun, M.Y.; Xu, Q.; Liu, C.H.; Min, L.H.; Li, H.P.; Liu, P.; Ding, X. Sorafenib inhibits transforming growth factor beta1-mediated epithelial-mesenchymal transition and apoptosis in mouse hepatocytes. *Hepatology* **2011**, *53*, 1708–1718.
